# Pathogenetic identification in ticks and yaks from Zoige County, China

**DOI:** 10.3389/fcimb.2024.1474519

**Published:** 2024-10-21

**Authors:** Yang Xiang, Liang He, Liangquan Zhu, Chendong Xiao, Yao Pan, Tianxiang Chen, Wei Zheng, Dongbo Yuan, Lili Hao

**Affiliations:** ^1^ College of Animal Science and Veterinary Medicine, Southwest Minzu University, Chengdu, China; ^2^ Animal Experiment Center, Institute of Animal Husbandry and Veterinary Science, Zhejiang Academy of Agricultural Sciences, Hangzhou, China; ^3^ Department of Bacterial Biologics, China Institute of Veterinary Drug Control (IVDC), Beijing, China; ^4^ Center for Animal Disease Control and Prevention in Xiangcheng County, Xiangcheng County Bureau of Agriculture, Animal Husbandry, Rural Affairs, and Science and Technology, Xiangcheng, China; ^5^ Public Health Department, Animal Husbandry Science Institute of Ganzi Tibetan Autonomous Prefecture, Kangding, China; ^6^ Center for Animal Disease Control and Prevention in Zoige County, Science and Technology and Agricultural and Animal Husbandry Bureau in Zoige County, Sichuan Ngawa Tibetan and Qiang Autonomous Prefecture, Zoige, China; ^7^ Guangxi Buffalo Research Institute, Chinese Academy of Agricultural Science, Nanning, China; ^8^ Public Health Department, Center for Animal Disease Control and Prevention in Sichuan Province, Chengdu, China

**Keywords:** *Anaplasma* spp., *Rickettsia* spp., Piroplasma, tick, yak (*Bos grunniens*)

## Abstract

**Background:**

Ticks represent a significant vector for the transmission of infectious diseases, with the prevalence of tick-borne diseases becoming a prominent global health concern in recent decades. *Anaplasma* spp., *Rickettsia* spp., and Piroplasma have been identified as significant pathogens with the potential to impact human and animal health. However, there is a dearth of data concerning the prevalence of these pathogens in the eastern Tibetan Plateau, China.

**Methods:**

In this study, a total of 643 *Dermacentor silvarum* and 314 *Haemaphysalis longicornis* were identified through the application of morphological and molecular identification techniques on 957 ticks collected from yaks in Zoige County. The assessed of *Anaplasma* spp., *Rickettsia* spp., *Theileria* spp., and *Babesia* spp. was assessed in 957 ticks and 96 blood samples collected from yaks.

**Results:**

Significant discrepancies were observed in the positivity rates for the four pathogens among the tick species and sampling sites. The identification of different species within the four pathogens was based on the analysis of the 16S rRNA of *Anaplasma* spp., the *ompA* and *ompB* genes of *Rickettsia* spp., and the 18S rRNA of *Theileria* spp. and *Babesia* spp. The prevalence ranges of the four pathogens are 9.9-50.2%, 29.5-100%, 16.2-46.4%, and 14.5-58.4%, respectively.

**Conclusion:**

In view of the growing zoonotic risks, further investigations into the prevalence of additional pathogens in ticks and animals, including livestock, in the eastern Tibetan Plateau, China, are essential.

## 1 Introduction

Ticks are significant vectors of infectious diseases, and they are recognized for their ability to sojourn on a variety of host species and transmit a variety of pathogens that can infect various vertebrate hosts, including humans. Anaplasmosis is the causative agent of tick-borne diseases, which have a significant impact on human and animal health (Buysse et al., 2024). The impact of anaplasmosis on the health and productivity of domestic animals has been well documented for over a century, and it remains a significant contributor to economic losses in the livestock farming industry. A minimum of seven species have been identified, including *A. marginale*, *A.* centrale, *A. ovis*, *A. phagocytophilum*, *A. bovis*, *A. capra*, and *A. platys*. It has been established through documented evidence that *A. ovis*, *A. phagocytophilum*, and *A. capra* have the potential to infect humans (Chochlakis et al., 2008; Lee et al., 2020; Li et al., 2015). Furthermore, novel *Anaplasma* species have been identified. In Japan, a potentially novel *Anaplasma* spp. was identified in a sika deer, exhibiting genetic divergence in the 16S rRNA, *gltA* and *groEL* genes from all known *Anaplasma* spp (Ybañez et al., 2012). The genus *Rickettsia* is an important vector-borne disease that has emerged or re-emerged globally and has increasingly posed a challenge to public health services. *Rickettsia* species are classified internationally into four groups: the spotted fever group (SFG), the transitional group (TRG), the ancestral group (AG), and the typhus group (TG). The SFG is the most diverse and geographically widespread group of known *Rickettsiae*. In China, a considerable proportion of SFG rickettsiosis have been identified as belonging to the *R. sibirica* group. Furthermore, additional *Rickettsia* species that are known to cause SFG rickettsiosis have also been identified, including *R. heilongjiangensis*, *R. sibirica*, *R. raoultii*, *R. slovaca*, *R. felis*, *R. aeschlimannii* and *R. massiliae* (Wei et al., 2015; Guo et al., 2016). The pathogens belonging to the genus *Theileria* and *Babesia* are among the most extensively researched parasites, due to the factors of their extensive geographical distribution, wide host range, and significant impact on public and animal health. Transmission occurs via the primary vectors, ixodid ticks, with disease outbreaks resulting in mortality, damage to hides, and poor production (Lempereur et al., 2017). Up to now, Theileria is only found in animals. In contrast to *Theileria*, three *Babesia* species have been identified as the causative agents of disease. These include *B. divergens*, *B. venatorum*, and *B. microti* with asymptomatic or mild but severe disease being predominantly observed in asplenic or immunocompromised individuals.

Nevertheless, there is a paucity of literature on the prevalence of these pathogens and their vectors in the eastern Tibetan Plateau, particularly in Zoige County (Wang et al., 2012). The Zoige region is home to the largest population of local livestock, namely yaks (*Bos grunniens*), which number approximately 800,000 individuals. These yaks represent the primary economic source for the local residents, providing dairy products, meat, and other by-products (Tang et al., 2019). The traditional lifestyle of the local population has resulted in a lack of timely deworming, which has led to the observation of severe tick infestation in yaks (Tang et al., 2019). Consequently, the objective of this study is to investigate the prevalence of *Anaplasma* spp., *Rickettsia* spp., *Theileria* spp. and *Babesia* spp. in ticks and yaks in Zoige County, providing preliminary data for the further control of transmission of diseases.

## 2 Materials and methods

### 2.1 Sample collection and identification of ticks

The research was conducted in 16 meadows situated in the villages of Jiangzha (longitude, 102.819; latitude, 34.176; altitude, 3373 m), Qiuji (longitude, 103.364; latitude 33.703; altitude, 2673 m), Hongxing (longitude, 102.734; latitude, 34.144; altitude, 3513 m), and Baxi (longitude, 103.240; latitude 33.634; altitude, 3212 m) in Zoige County, Sichuan Province, China, from 7 April to 26 September 2020. Approximately three to five yaks were selected from each meadow, with five to six ticks collected from each yak. The feeding ticks were identified based on their morphological characteristics, with the aid of standard taxonomic keys (Deng and Jiang, 1991). Prior to polymerase chain reaction (PCR) amplification, during which the COI gene was targeted (Folmer et al., 1994), the ticks were stored in 70% ethanol at 4°C. In addition, a total of 96 blood samples were obtained from the yaks, with four yaks sampled from each of the six randomly selected meadows in each village. All blood samples were stored at -20°C until further use. Further details regarding the collection of tick and blood samples can be found in the **Supplementary Materials** (**Supplementary Figure 1**).

### 2.2 DNA extraction and PCR amplification

Each tick was subjected to individual DNA extraction using a DP304 TIANamp Genomic DNA Kit (TIANGEN Biotech Co., Ltd., Beijing, China), in accordance with the manufacturer’s instructions. Genomic DNA was extracted from all blood samples using an EE121-11 Blood Genomic DNA Kit (Transgen Biotech Co., Ltd., Beijing, China). All genomic DNAs were stored at -20°C until analysis was conducted. Nested polymerase chain reaction (nPCR) amplification was conducted in accordance with previously published criteria targeting the 16S ribosomal RNA (rRNA) of *Anaplasma* spp., the 18S rRNA of *Theileria* spp. and *Babesia* spp., and the outer membrane protein A (*ompA*) and the outer membrane protein B (*ompB*) genes of *Rickettsia* spp (Raoult et al., 2005). The primer sequences are provided in **Table 1**. The initial screening of ticks and blood samples was conducted using the *Rickettsia* spp. *ompA* gene. Subsequently, samples that tested positive for the *ompA* gene were subjected to further screening for the *ompB* gene in accordance with the established criteria for *Rickettsia* species (Oteo et al., 2006; Fernández et al., 2013). The reaction mixture comprised 2 μL of template DNA, 12.5 μL of 2 × PCR mix (TransGen Biotech Co., Ltd., Beijing, China, Cat No: AS111), and 20 pmol of each primer (Sangon Biotech Co., Ltd., Shanghai, China). The initial denaturation was performed at 95°C for three minutes, 40 cycles of denaturation at 94°C for 30 seconds, annealing at 55°C for 30 seconds, and elongation at 72°C for 55 seconds. Subsequently, a final extension step was conducted at 72°C for seven minutes. The PCR products were electrophoresed on a 1.2% agarose gel mixed with Liuyi (Beijing Liuyi Biotechnology Co., Ltd., China), and the expected bands were visualized using a UV transilluminator. The observed bands were purified using the QIAquick Gel Extraction Kit and sent for sequencing (Sangon Biotech Shanghai Co., Ltd.).

**Table 1:** Primer sequences used for tick, *Anaplasma* spp., *Theileria* spp., *Babesia* spp. and *Rickettsia* spp. Identification.

Target gene	Primer sequence (5’-3’)	Product (bp)
*COI*	LCO1490: GGTCAACAAATCATAAAGATATTGG HCO2198: TAAACTTCAGGGTGACCAAAAAATCA	658
16S rRNA (*Anaplasma* spp.)	EE1: TCCTGGCTCAGAACGAACGCTGGCGGC EE2: AGTCACTGACCCAACCTTAAATGGCTG	1433
	EE3: TACCTCTGTGTTGTAGCTAACGC EE4: CTTGCGACATTGCAACCTATTGT	426
*OmpA* (*Rickettsia* spp.)	Rr190k.71p: TGGCCAATATTTCTCCAAAA Rr190k.720n: TGCATTTGTATTACCTATTGT	650
	Rr190.70p: ATGGCGAATATTTCTCCAAAA Rr190.602n: AGTGCAGCATTCGCTCCCCCT	530
*OmpB* (*Rickettsia* spp.)	OmpB.4362: GTCAGCGTTACTTCTTCGATGC OmpB.4836: CCGTACTCCATCTTAGCATCAG	475
	OmpB.4496: CCAATGGCAGGACTTAGCTACT OmpB.4762: AGGCTGGCTGATACACGGAGTAA	267
18S rRNA (*Theileria* spp.)	LTF: GATAACCGTGCTAATTGTAGG LTR: ATCGTCTTCGATCCCCTAACT	843
	LTF2: AATTGTAGGGCTAATACATGTTCG LTR2: GAAAACATCCTTGGCAAATGCTTTCGC	760
18S rRNA (*Babesia* spp.)	B1200F: GGAATGATGGYGACBTAAACCCTCA B1200R: CTTCCCTAGGCNAARCCGACGAAT	1200
	B1200F: GGAATGATGGYGACBTAAACCCTCA B1000R: GGCATTCCTCGTTCATGATTTAG	1000

### 2.3 Molecular phylogenetic analyses

The sequences were subjected to analysis and comparison using the DNASTAR v.7.1.0 software. The nucleotide sequences were analyzed using the BLAST tool, as previously described by Sayers et al. (2021), in order to compare them with sequences deposited in GenBank (Benson et al., 2002). Phylogenetic trees were constructed using the Neighbor-Joining method in MEGA 6 software, based on the *COI*, 16S rRNA, 18S rRNA, *ompA* and *ompB* genes, respectively. The evolutionary distance was calculated using the Kimura 2-parameter method with 1,000 bootstrap replicates.

### 2.4 Statistics

The prevalence of *Anaplasma* spp., *Rickettsia* spp. *Theileria* spp., and *Babesia* spp. was found to be statistically significant (*p*-value< 0.01) when analyzed according to the different sampling locations, tick species, or yaks, as determined by a Pearson Chi-square test, conducted using SPSS 19.0 (IBM, New York, USA).

## 3 Results

### 3.1 Species identification and distribution of ticks collected from yaks

**Figure 1:**
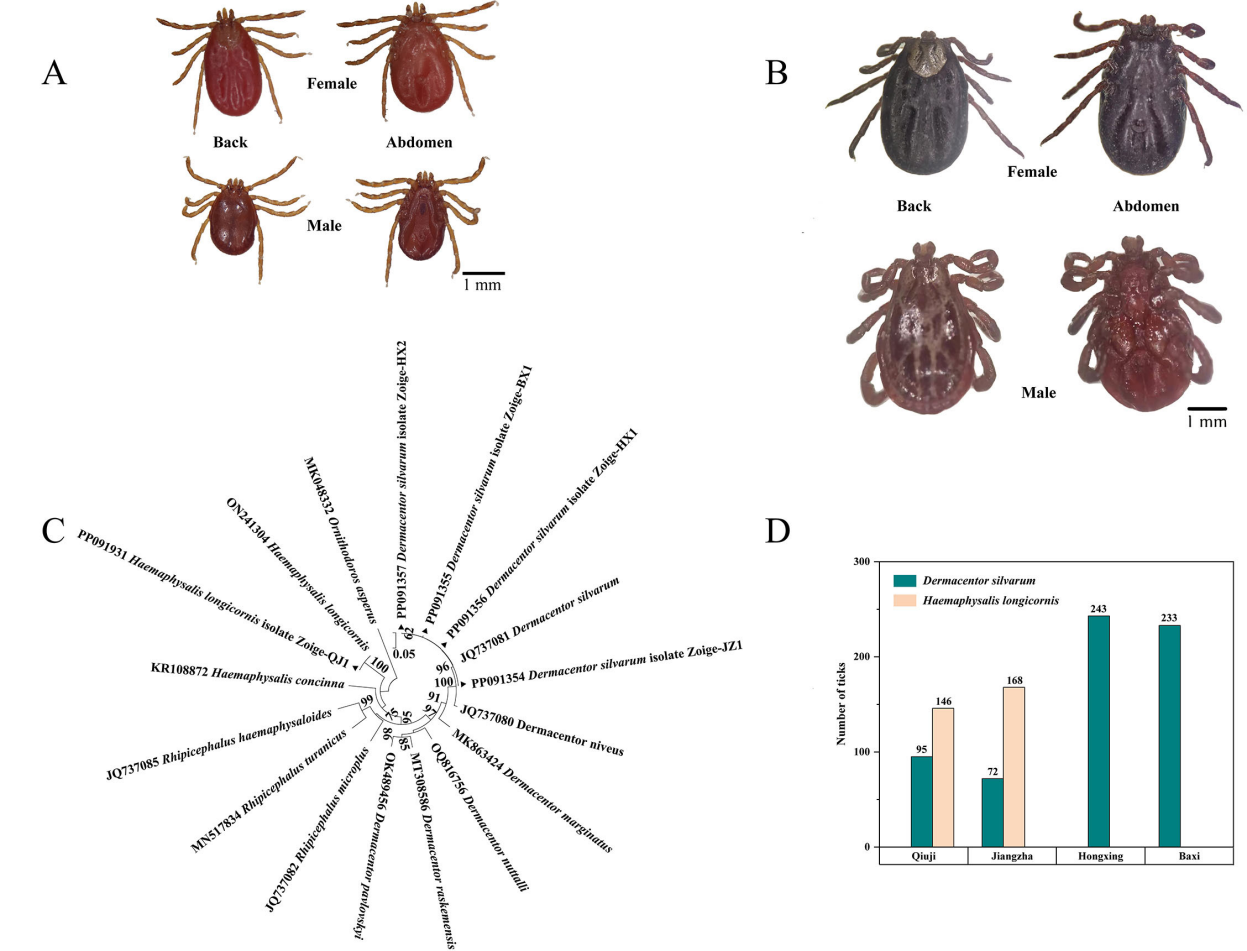
Species identification and distribution of ticks: **(A)** the morphological characteristics of ticks belonging to the *H. longicornis* (Collected from Qiuji in 2020); **(B)** the morphological characteristics of ticks belonging to the *D. silvarum* (Collected from Jiangzha in 2020); **(C)** Molecular identification and phylogenetic analysis of ticks base on the *COI* gene. MEGA 6 software was used to infer the tree by using the neighbor-joining method with Kimura’s two-parameter model. Support for each branch, as deter-mined from 1000 bootstrap samples, is indicated by percentages at nodes (only values > 60% are indicated). Triangle and serial number in the figure indicate the distinct sequence in our study. Bar, 0.05 substitutions per nucleotide position; **(D)** distribution characteristics of ticks in different villages.

A total of 957 adult ticks were collected from the villages of Jiangzha, Qiuji, Hongxing, and Baxi in Zoige County, Sichuan Province, China. The number of ticks collected from each of the villages of Qiuji, Jiangzha, Hongxing, and Baxi was 241, 240, 243, and 233, respectively. A total of 643 ticks were preliminarily identified as *Dermacentor silvarum*, while a total of 314 ticks were identified as *Haemaphysalis longicornis* based on the morphological characteristics of the ticks (**Figures 1A, B**). In addition, a total of five distinct *COI* sequences (denoted as *D. silvarum* JZ, *D. silvarum* BX, *D. silvarum* HX1, *D. silvarum* HX2, and *H. longicornis*) were identified through multiple sequence alignment. The combination of morphological and *COI* gene identification confirmed the presence of two distinct tick species, belonging to the *D. silvarum* and *H. longicornis* (**Figure 1C**). Consequently, the ticks belonging to *D. silvarum* were present in all four villages. However, another species was only identified in Qiuji village (146) and Jiangzha village (168). The prevalence of *D. silvarum* was found to be higher in Qiuji village (39.4%) than in Jiangzha village (30.0%) (**Figure 1D**).

### 3.2 Pathogens and occurrence in ticks and yaks

#### 3.2.1 Pathogens in ticks

A total of four pathogens, including *Anaplasma* spp., *Rickettsia* spp., *Theileria* spp., and *Babesia* spp., were identified in ticks from four villages, with an average positive rate of 29.8% (168/957), 64.6% (618/957), 30.1% (288/957) and 37.6% (360/957), respectively (**Table 2**). All four pathogens were identified in both *D. silvarum* and *H. longicornis*. Significant differences were observed in the positive rates for *Anaplasma* spp. (*χ^2^* = 12.506, *df* = 1), *Rickettsia* spp. (*χ^2^* = 138.55, *df* = 1, *P* < 0.01), *Theileria* spp. (*χ^2^* = 42.625, *df* = 1, *P* < 0.01), and *Babesia* spp. (*χ^2^* = 56.993, *df* = 1, *P* < 0.01) among the two tick species. The infection rates of *Rickettsia* spp. in ticks were found to be significantly higher than that of the other pathogens (*χ^2^* = 322.109, *df* = 3, *P* < 0.01). The infection rates of *Rickettsia* spp. were 77.3% and 38.5% in *H. longicornis* and *D. silvarum*, respectively. Significant differences were observed in the infection rates of *Anaplasma* spp., *Rickettsia* spp., *Theileria* spp., and *Babesia* spp. in ticks across the four villages (*χ^2^* = 322.109, *df* = 3, *P* < 0.01). In general, the highest positive rates for *Anaplasma* spp., *Rickettsia* spp., *Theileria* spp., and *Babesia* spp. were observed in Baxi village, with positive rates of 50.2% (*χ^2^* = 112.213, *df* = 3, *P* <, *P* < 0.01), 100.0% (*χ^2^* = 154.454, *df* = 3, *P* < 0.01), 46.4% (*χ^2^* = 23.179, *df* = 3, *P* < 0.01) and 58.4% (*χ^2^* = 67.646, *df* = 3, *P* < 0.01), respectively. The lowest positive rates for *Anaplama* spp., *Rickettsia* spp., *Theileria* spp., and *Babesia* spp. were observed in Qiuji village, with positive rates of 10.0%, 29.5%, 16.2%, and 14.5%, respectively.

**Table 2:** Pathogens and occurrence in ticks from four villages.

Location	Pathogen	Qiuji	Jiangzha	Hongxing	Baxi	Total
*Dermacentor silvarum*	*Anaplasma* spp.	17.9% (17/95)	13.9% (10/72)	9.9% (24/243)	**50.2%** (117/233)	26.1% (168/643)
	*Rickettsia* spp.	52.6% (50/95)	94.4% (68/72)	60.1% (146/243)	**100%** (233/233)	77.3% (497/643)
	*Theileria* spp.	20.0% (19/95)	27.8% (20/72)	37.0% (90/243)	**46.4%** (108/233)	36.9%(237/643)
	*Babesia* spp.	15.8% (15/95)	23.6% (17/72)	52.3% (127/243)	**58.4%** (136/233)	45.9% (295/643)
*Haemaphysalis longicornis*	*Anaplasma* spp.	4.8% (7/146)	65.5% (110/168)	0	0	37.3% (117/314)
	*Rickettsia* spp.	14.4% (21/146)	59.5% (100/168)	0	0	38.5% (121/314)
	*Theileria* spp.	13.7% (20/146)	18.5% (31/168)	0	0	16.2% (51/314)
	*Babesia* spp.	13.7% (20/146)	26.8% (45/168)	0	0	20.7% (65/314)
Total	*Anaplasma* spp.	10.0% (24/241)	50.0% (120/240)	9.9% (24/243)	50.2% (117/233)	29.8% (285/957)
	*Rickettsia* spp.	29.5% (71/241)	70.0% (168/240)	60.1% (146/243)	100% (233/233)	64.6% (618/957)
	*Theileria* spp.	16.2% (39/241)	21.3% (51/240)	37.0% (90/243)	46.4% (108/233)	30.1% (288/957)
	*Babesia* spp.	14.5% (35/241)	25.8% (62/240)	52.3% (127/243)	58.4% (136/233)	37.6% (360/957)

Bold means extremely significant difference between the treatments in the same line (P< 0.01).

#### 3.2.2 Pathogens in yaks

The infection rates of *Anaplasma* spp. in yaks collected from Qiuji, Jiangzha, Hongxing, and Baxi village were 41.7%, 41.7%, 58.3%, and 70.8%, respectively (**Table 3**). In contrast, *Rickettsia* positive yaks were only found in Qiuji, and had an infection prevalence of 12.5%. *Theileria* positive yaks collected from Qiuji, Jiangzha, Hongxing, and Baxi village were 50.0%, 37.5%, 62.5%, and 58.3%, respectively. In contrast, *Babesia* positive yaks in the same villages were 37.5%, 33.3%, 54.2%, and 70.8%, respectively.

**Table 3:** Pathogens and occurrence in yaks from four villages.

Location	*Anaplasma* spp.	*Rickettsia* spp.	*Theileria* spp.	*Babesia* spp.
Qiuji	41.7%(10/24)	12.5%(3/24)	50.0%(12/24)	37.5%(9/24)
Jiangzha	41.7%(10/24)	0(0/24)	37.5%(9/24)	33.3%(8/24)
Hongxing	58.3%(14/24)	0(0/24)	58.3%(14/24)	54.2%(13/24)
Baxi	**70.8%(17/24)**	0(0/24)	**62.5%(15/24)**	**70.8%(17/24)**
Total	53.1%(51/96)	**3.1%(3/96)**	52.1%(50/96)	49.0%(47/96)
*χ^2^*	5.814	9.290	3.506	8.462
*P*	0.121	0.026	0.320	0.037

Bold means extremely significant difference between the treatments in the same line (P< 0.01).

### 3.3 Phylogeny of *Anaplasma* spp., *Rickettsia* spp. *Theileria* spp., and *Babesia* spp.

#### 3.3.1 *Anaplasma* spp. in ticks and yaks

**Figure 2:**
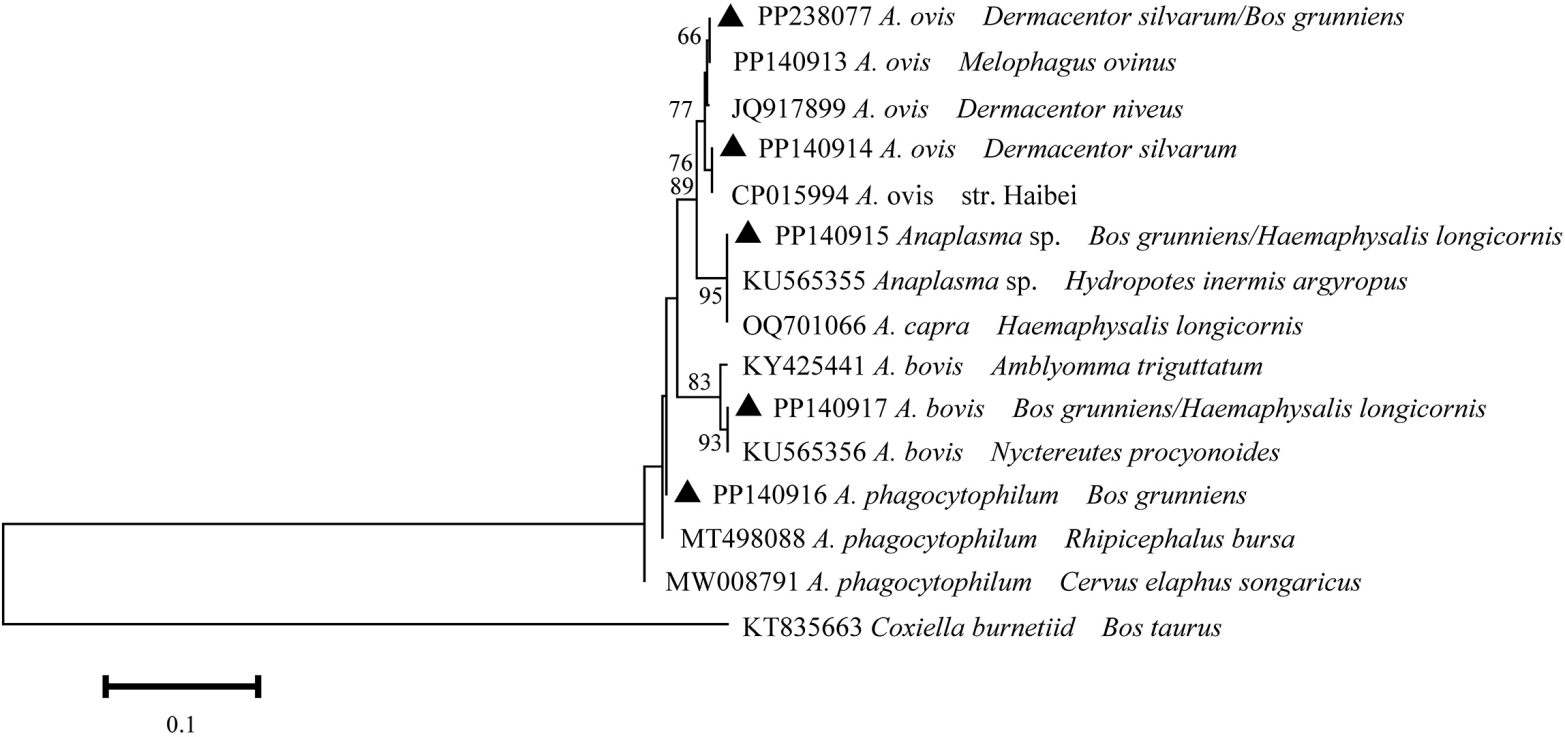
Phylogeny of *Anaplasma* spp. in ticks and yaks. MEGA 6 software was used to infer the tree by using the neighbor-joining method with Kimura’s two-parameter model. Support for each branch, as deter-mined from 1000 bootstrap samples, is indicated by percentages at nodes (only values > 60% are indicated). Triangle and serial number in the figure indicate the distinct sequence in our study. Bar, 0.1 substitutions per nucleotide position.

A total of five distinct 16S rDNA sequences were identified from the positively identified samples through multiple sequence alignment. The aforementioned sequences were designated as PP238077, PP140914, PP140915, PP140916, and PP140917. The PP238077, PP140914, PP140915, PP140917 and PP140916 sequences exhibited a high degree of similarity (99.4-100%) to *A. ovis* (PP140913), *A. ovis* str. Haibei (CP015994), *A. capra* (OQ701066), *A. bovis* (KY425441), and *A. phagocytophilum* (MT498088) (**Figure 2**). It can thus be posited that the *Anaplasma* spp. may be implicated in five species (*A. capra*, *A. bovis*, *A. ovis*, *A. phagocytophilum*, and *A.* sp.), which have been identified in infected ticks and yaks.

#### 3.3.2 *Rickettsia* spp. in ticks and yaks

**Figure 3:**
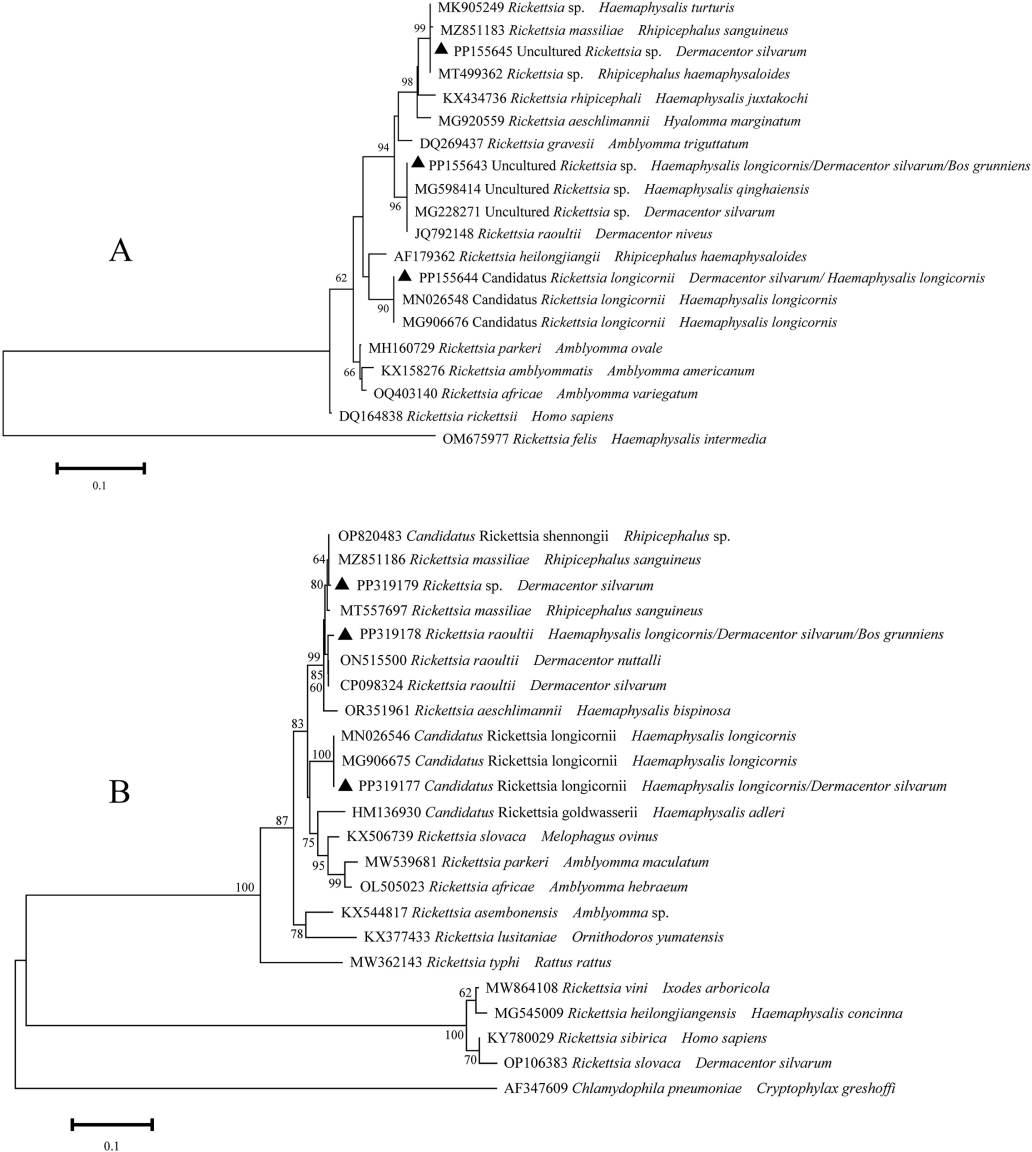
Phylogeny of *Rickettsia* spp. in ticks and yaks, **(A)** *ompA* amplicons; **(B)** *ompB* amplicons. MEGA 6 software was used to infer the tree by using the neighbor-joining method with Kimura’s two-parameter model. Support for each branch, as deter-mined from 1000 bootstrap samples, is indicated by percentages at nodes (only values > 60% are indicated). Triangle and serial number in the figure indicate the distinct sequence in our study. Bar, 0.1 substitutions per nucleotide position.

A total of six distinct sequences of *ompA* and *ompB* amplicons from the samples that had been identified as positive were identified through multiple sequence alignments. The aforementioned sequences were subsequently designated as PP155643-PP155645, PP319177-PP319179. The PP155643-PP155645 sequences were closely related to *R. raoultii* (JQ792148), Candidatus *R. longicornii* (MN026548), and *R. massiliae* (MZ851183) (**Figure 3**). These sequences exhibited 99.8-100% sequence identity. With regard to the *ompB* gene, the sequences PP319177- PP319179 exhibited 100%, 99.4%, and 99.8% identical to Candidatus *R. longicornii* (MN026546), *R. raoultii* (ON515500), and *R. massiliae* (MZ851186), respectively. It can thus be posited that the *Rickettsia* spp. may be implicated in three species (*R. massiliae*, *R. raoultii*, and Candidatus *R. longicornii*), which have been identified in infected ticks and yaks.

#### 3.3.3 *Theileria* spp. in ticks and yaks

**Figure 4:**
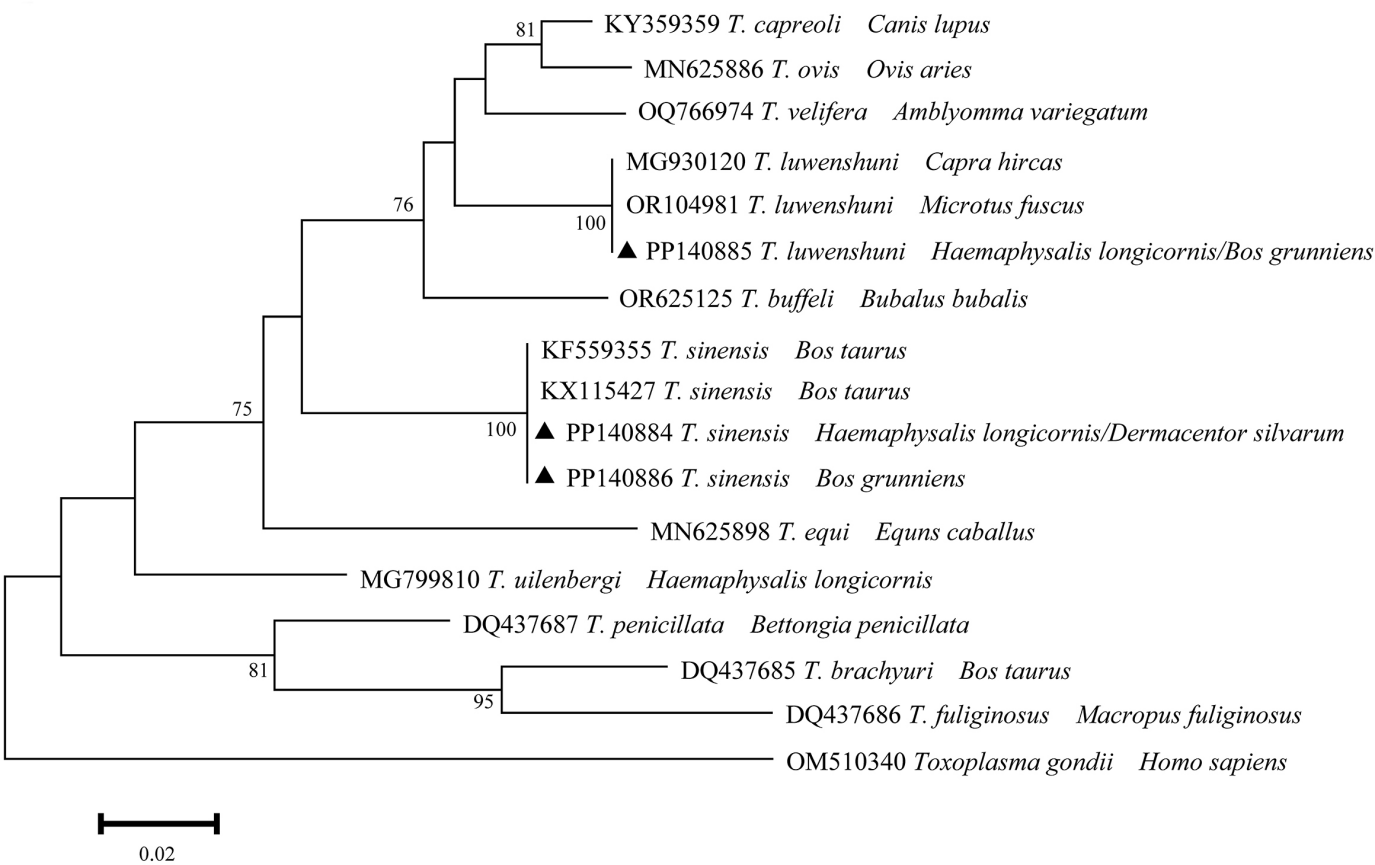
Phylogeny of *Theileria* spp. in ticks and yaks. MEGA 6 software was used to infer the tree by using the neighbor-joining method with Kimura’s two-parameter model. Support for each branch, as deter-mined from 1000 bootstrap samples, is indicated by percentages at nodes (only values > 60% are indicated). Triangle and serial number in the figure indicate the distinct sequence in our study. Bar, 0.02 substitutions per nucleotide position.

A total of three distinct sequences of 18S rRNA of *Theileria* spp. were identified through multiple sequence alignments from the samples that had been positively identified. The aforementioned sequences were designated as PP140884-PP140886. The unique sequences (PP140884 and PP140886) exhibited 100% identity with KF559355 and KX115427 (*T*. *sinensis*), and PP140885 exhibited 100% identity with MG930120 and OR104981 (*T*. *luwenshuni*) (**Figure 4**). It can thus be posited that *Theileria* spp. may be implicated in two species (*T*. *sinensis* and *T*. *luwenshuni*), which have been identified in infected ticks and yaks.

#### 3.3.4 *Babesia* spp. in ticks and yaks

**Figure 5:**
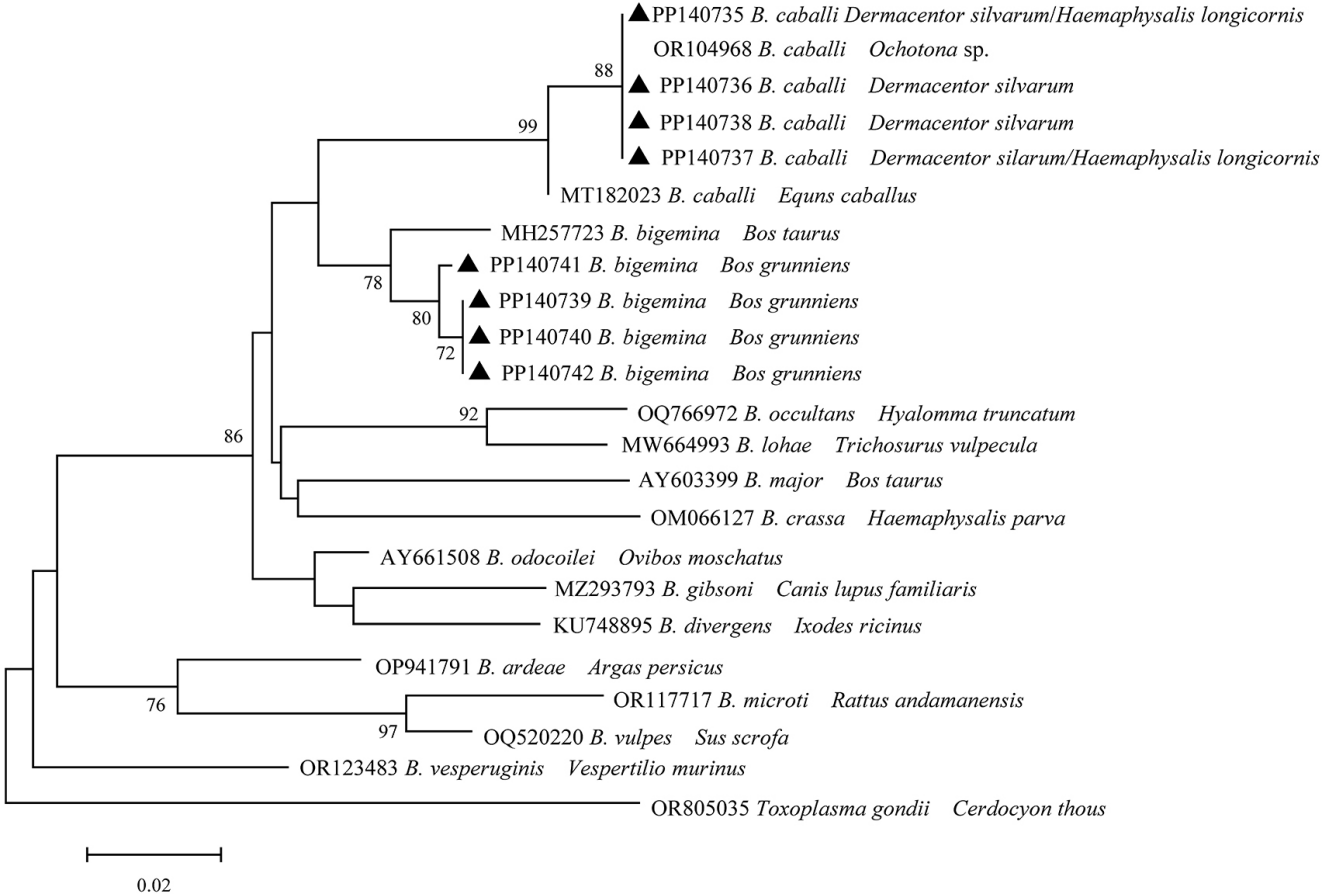
Phylogeny of Babesia spp. in ticks and yaks. MEGA 6 software was used to infer the tree by using the neighbor-joining method with Kimura’s two-parameter model. Support for each branch, as deter-mined from 1000 bootstrap samples, is indicated by percentages at nodes (only values > 60% are indicated). Triangle and serial number in the figure indicate the distinct sequence in our study. Bar, 0.02 substitutions per nucleotide position.

A total of eight distinct sequences of 18S rRNA of *Babesia* spp. from the samples that had been positively identified were identified through multiple sequence alignment. The aforementioned sequences were subsequently designated as PP140735, PP140736, PP140737, PP140738, PP140739, PP140740, PP140741, and PP140742. Four distinct sequences (PP140735- PP140738) exhibited 98.6-99.3% identity to *B. caballi* (OR104968) with 100% coverage (**Figure 5**). Additionally, four distinct sequences (PP140739-PP140742) were identified within the same cluster with *B*. *bigemina* (MH257723). It can thus be concluded that *Babesia* spp. may be involved in two species (*B*. *bigemina* and *B. caballi*), which have been identified in infected ticks and yaks.

## 4 Discussion

A total of 957 ticks infesting yaks were collected from the villages of Jiangzha, Qiuji, Hongxing, and Baxi in Zoige County, Sichuan Province, China. The combination of morphological and molecular identification techniques facilitates more precise identification of ticks, representing the most prevalent approach to parasite identification in current practice (Estrada-Pena et al., 2017). In this study, two species of ticks were identified: *D. silvarum* and *H. longicornis*. *Dermacentor silvarum* was the dominant species in four villages, whereas *H. longicornis* was only observed in Jiangzha and Qiuji. Previously, *D. silvarum* was documented in the northern hemisphere, with a range extending from 22° N to 57° N latitude (Guo et al., 2021). *Dermacentor silvarum* has been identified in 11 provinces, three autonomous regions, and one municipality of China, with a total of 290 counties and 34 prefectures (Guo et al., 2021). The initial report of the detection of *D. silvarum* in Sichuan province was published (Liu, 2021). As has been previously documented, *D. silvarum* is typically found near mountain ranges and exhibits a preference for deciduous and coniferous forests, as well as cultivated and shrubby vegetations (Guo et al., 2021). In this study, *D. silvarum* was identified at altitudes ranging from 2673 to 3513 meters and was observed to be located in grassland areas devoid of mountainous terrain in the vicinity. This constitutes a valuable addition to the known habitat of *D. silvarum*. Conversely, *H. longicornis* has been identified across a range of latitudes, from 18° to 53° in the Northern Hemisphere and from 16° to 45° in the Southern Hemisphere (Zhao et al., 2020). The species has been identified in all provinces of China (Zhao et al., 2020). In contrast to *D. silvarum*, *H. longicornis* was observed only in Qiuji and Jiazhang at altitudes below 3, 000 m. It was hypothesized that the most suitable habitat for *H. longicornis* would be coastal areas, with eastern North America identified as a particularly promising location (Namgyal et al., 2020). Nevertheless, our research has identified grassland as a suitable habitat for *H. longicornis*. This also represents a valuable addition to the habitat of *H. longicornis*. The primary hosts of *D. silvarum* were primarily domestic animals, including cattle, goats, and sheep (Guo et al., 2021). In contrast, a total of 77 species of animals have been identified as potential hosts of *H. longicornis* (Zhao et al., 2020). The findings of this research indicate that the yak is the optimal host for two tick species, with severe tick infestation frequently observed in Zoige. The Zoige County region is home to a considerable number of yaks, sheep, horses, *Marmota himalayana*, *Lepus* spp., *Myospalax* spp. and *Ochotona* spp., with a wide geographical range and a notable distribution (Zhou et al., 2021). To obtain more detailed information on tick hosts, it is recommended that tick samples should be collected from a variety of sources, including other animals, vegetation, and even the local community. The infection risk for humans and animals can be estimated by dragging blankets across grasslands to capture and calculate the numbers of unfed ticks in a given area (Edwards et al., 2022). The paucity of clinical cases reported or recorded can be attributed to the traditional lifestyle of the local community, particularly the practice of herding (Tang et al., 2019). This lifestyle renders them less likely to seek medical attention from a hospital unless they have a serious issue.

Ticks play a significant role in the transmission and propagation of a diverse array of serious zoonotic diseases, acting as vectors and reservoirs for a multitude of pathogens (Athni et al., 2021). A total of four genera of pathogens were identified in ticks and yaks: *Anaplasma* spp., *Rickettsia* spp., *Theileria* spp., and *Babesia* spp., with at least two species detected in each genus, which represents a significant public health concern (Livengood et al., 2020). The extant literature indicates that *Anaplasma* spp. can be classified into several distinct categories, including *A. ovis*, *A. phagocytophilum*, *A. capra*, *A. marginale*, *A. platys*, *A. bovis* and *A. centrale*. Of these, *A. ovis*, *A. phagocytophilum*, and *A. capra* have been reported to infect humans (Chochlakis et al., 2008; Lee et al., 2020; Li et al., 2015). In this study, five species were identified in infected ticks and yaks: *A. capra*, *A. bovis*, *A. ovis*, *A. phagocytophilum*, and *A.* spp. The prevalence of *Rickettsiae* spp. in *D. silvarum* was higher than that in *H. longicornis*. The highest prevalence was observed in Baxi, followed by Jiangzha, Hongxing, and Qiuji. In accordance with the established criteria for determining the *Rickettsiae* spp., three species (*R. massiliae*, *R. raoultii*, and Candidatus *R. longicornii*) were identified in infected ticks and yaks. The first isolation of *R. raoultii* from ticks was reported in Russia (Rydkina et al., 1999). Subsequent studies have identified the presence of this bacterium in at least 26 tick species belonging to seven genera, including *Dermacentor* (Jia et al., 2014), *Haemaphysalis* (Li et al., 2018; Zheng et al., 2018)*, Amblyomma* (Parola et al., 2013), *Rhipicephalus* (Liu et al., 2018), *Ixodes* (Rar et al., 2017; Shpynov et al., 2009), and *Hyalomma* (Yin et al., 2018). *Rickettsia raoultii* has been predominantly identified in *Dermacentor* spp. ticks across multiple countries in Europe (Mediannikov et al., 2008; Wang et al., 2012; Foldvari et al., 2013; Spitalska et al., 2012). Furthermore, *R. massiliae* was identified in ticks collected from yaks, indicating that the local area is a risk area for *R. massiliae* infection and that prevention and control of local ticks should be strengthened. *Theileria sinensis* was initially identified in 1995 by Bai Qi from cattle in the northwestern region of China (Bai et al., 1997). *Theileria sinensis* exhibits relatively weak pathogenicity, and is primarily reported in Asia, including China, Japan, and the Korean Peninsula. In 2020, the DNA of *T. sinensis* was identified in yaks in the neighboring regions of Hongyuan and Aba in Sichuan Province, China (Hao et al., 2020). *Theileria luwenshuni* was initially identified in sheep and goats, exhibiting high pathogenicity. It is widely distributed throughout most parts of China and is primarily transmitted by both *H. longicornis* and *H. qinghaiensis* (Li et al., 2007). Of the two *Babesia* species detected, *B. bigemina* is a globally distributed agent of bovine babesiosis. The current literature indicates that *B. bigemina* is present in at least five tick species, including *R. microplus*, *R. decoloratus*, *R. annulatus*, *R. geigyi* and *R. evertsi*. In China, *B. bigemina* has been predominantly documented in ticks (*R. microplus*) and domestic animals across numerous provinces including Qinghai, Gansu, Guangxi, Chongqing, Liaoning, Yunnan, Shandong, Henan, Hubei and Xinjiang (He et al., 2021). *B. caballi* is the pathogen responsible for equine babesiosis, which affects horses, donkeys and mules (Cui et al., 2024). It is primarily transmitted by ticks including *D. silvarum*, *D. ralbipictus*, *D. nitens*, *D. reticulates*, and *H. truncatum* (Zhang et al., 2021). The subsequent step is to investigate local horses, donkeys, mules, and other equine animals to ascertain the prevalence of *B. caballi* in the area.

## 5 Conclusions

In the course of this study, two species of ticks (*D. silvarum* and *H. longicornis*) were identified through the application of morphological and molecular identification techniques. Furthermore, the phylogeny of these pathogens was explored encompassing *Anaplasma* spp., *Rickettsia* spp., *Theileria* spp., and *Babesia* spp. To gain a more comprehensive understanding of the infection risk for humans and animals, it would be beneficial to conduct a more detailed study of pathogens in other livestock and wildlife hosts from Zoige County in the future.

## Data Availability

The original contributions presented in the study are included in the article/**Supplementary Material**. Further inquiries can be directed to the corresponding author.

## Supplementary material

Supplementary Figure 1 Tick sampling information.

## References

[B1] Athni T. S. Shocket M. S. Couper L. I. Nova N. Caldwell I. R. Caldwell J. M.. (2021). The influence of vector-borne disease on human history: socio-ecological mechanisms. Ecol. Lett. 24, 829–846. doi: 10.1111/ele.13675 33501751 PMC7969392

[B2] Bai Q. Liu Y. G. Han G. F. (1997). An unidentified species of *Theileria* infective for cattle discovered in China. Trop. Anim. Health Pro 29, 43S. doi: 10.1007/BF02632917 9512745

[B3] Benson D. A. Karsch-Mizrachi I. Lipman D. J. Ostell J. Rapp B. A. Wheeler D. L. (2002). GenBank. Nucleic Acids Res. 30, 17–20. doi: 10.1093/nar/30.1.17 11752243 PMC99127

[B4] Buysse M. Koual R. Binetruy F. De T. B. Baudrimont X. Garnier S.. (2024). Detection of *Anaplasma* and *Ehrlichia* bacteria in humans, wildlife, and ticks in the Amazon rainforest. Nat. Commun. 15, 3988. doi: 10.1038/s41467-024-48459-y 38734682 PMC11088697

[B5] Chochlakis D. Koliou M. Ioannou I. Tselentis Y. Psaroulaki A. (2008). Kawasaki disease and *Anaplasma* sp. infection of an infant in Cyprus. Int. J. Infect. Dis. 13, e71–e73. doi: 10.1016/j.ijid.2008.08.001 18848483

[B6] Cui Y. Y. Cao M. Y. Yu F. C. Zhao A. Y. Tao D. Y. Zhu T. T.. (2024). Molecular detection of piroplasms in domestic donkeys in Xinjiang, China. Vet. Med. Sci. 10, e1468. doi: 10.1002/vms3.v10.4 38879882 PMC11180456

[B7] Deng G. F. Jiang Z. J. (1991). Economic Insect Fauna of China. Ixodes [M] (Beijing: Science Press).

[B8] Edwards C. D. Campbell H. (2022). Sampling implications of variation in daily activity of the sheep tick, Ixodes ricinus at a coastal grassland site in the UK. Med. Vet. Entomol. 36, 127–132. doi: 10.1111/mve.12543 34338344

[B9] Estrada-Pena A. Amico G. Palomar A. M. Dupraz M. Fonville M. Heylen D.. (2017). A comparative test of ixodid tick identification by a network of European researchers. Ticks Tick Borne Dis. 8, 540–546. doi: 10.1016/j.ttbdis.2017.03.001 28320640

[B10] Fernández D. M. I. G. Ruiz-Fons F. De-la F. G. Mangold A. J. Gortázar C. De-la F. J. (2013). Spoted Fever Group Rickettsiae in questing ticks, central Spain. Emerg. Infect. Dis. 19, 1163–1165. doi: 10.3201/eid1907.130005 23763913 PMC3713984

[B11] Foldvari G. Rigo K. Lakos A. (2013). Transmission of *Rickettsia slovaca* and *Rickettsia raoultii* by male *Dermacentor marginatus* and *Dermacentor reticulatus* ticks to humans. Diagn. Microbiol. Infect. Dis. 76, 387–389. doi: 10.1016/j.diagmicrobio.2013.03.005 23602788

[B12] Folmer O. Black M. Hoeh W. Lutz R. Vrijenhoek R. (1994). DNA primers for amplification of mitochondrial cytochrome c oxidase subunit I from diverse metazoan invertebrates. Mol. Mar. Biol. Biotech. 3, 294–9. doi: 10.4028/www.scientific.net/DDF.7.460 7881515

[B13] Guo L. P. Jiang S. H. Liu D. Wang S. W. Chen C. F. Wang Y. Z. (2016). Emerging spotted fever group rickettsiae in ticks, northwestern China. Ticks Tick Borne Dis. 7, 1146–1150. doi: 10.1016/j.ttbdis.2016.08.006 27554852

[B14] Guo W. B. Shi W. Q. Wang Q. Pan Y. S. Chang Q. C. Jiang B. G.. (2021). Distribution of *Dermacentor silvarum* and associated pathogens: meta-analysis of global published data and a field survey in China. Int. J. Env. Res. Pub He 18, 4430. doi: 10.3390/ijerph18094430 PMC8122522 33921917

[B15] Hao L. Yuan D. Li S. Jia T. Guo L. Hou W.. (2020). Detection of *Theileria* spp. in ticks, sheep keds (*Melophagus ovinus*), and livestock in the eastern Tibetan Plateau, China. Parasitol. Res. 119, 2641–2648. doi: 10.1007/s00436-020-06757-6 32556503

[B16] He L. Bastos R. G. Sun Y. Hua G. Guan G. Zhao J.. (2021). Babesiosis as a potential threat for bovine production in China. Parasite Vector 14, 460. doi: 10.1186/s13071-021-04948-3 PMC8425137 34493328

[B17] Jia N. Zheng Y. C. Ma L. Huo Q. B. Ni X. B. Jiang B. G.. (2014). Human infections with *Rickettsia raoultii*, China. Emerg. Infect. Dis. 20, 866–868. doi: 10.3201/eid2005.130995 24750663 PMC4012798

[B18] Lee S. H. Shin N. R. Kim C. M. Park S. Yun N. R. Kim D. M.. (2020). First identification of *Anaplasma phagocytophilum* in both a biting tick *Ixodes nipponensis* and a patient in Korea. BMC Infect. Dis. 20, 826. doi: 10.1186/s12879-020-05522-5 33176719 PMC7656494

[B19] Lempereur L. Beck R. Fonseca I. Marques C. Duarte A. Santos M.. (2017). Guidelines for the detection of *Babesia* and *Theileria* parasites. Vector Borne Zoonot 17, 51–65. doi: 10.1089/vbz.2016.1955 28055573

[B20] Li H. Zhang P. H. Huang Y. Du J. Cui N. Yang Z. D.. (2018). Isolation and identification of *Rickettsia raoultii* in human cases: a surveillance study in 3 medical centers in China. Clin. Infect. Dis. 66, 1109–1115. doi: 10.1093/cid/cix917 29069294

[B21] Li H. Zheng Y. C. Ma L. Jia N. Jiang B. G. Jiang R. R.. (2015). Human infection with a novel tick-borne *Anaplasma* species in China: a surveillance study. Lancet Infect. Dis. 15, 663–670. doi: 10.1016/S1473-3099(15)70051-4 25833289

[B22] Li Y. Luo J. Liu Z. Guan G. Gao J. Ma M.. (2007). Experimental transmission of *Theileria* sp. (China 1) infective for small ruminants by *Haemaphysalis longicornis* and *Haemaphysalis qinghaiensis* . Parasitol. Res. 101, 533–538. doi: 10.1007/s00436-007-0509-8 17370090

[B23] Liu C. C. (2021). Identification of tick species and molecular detection of Spotted fever group, Bartonella and Anaplasma in ticks and blood from Yak in Ruoergai County of Sichuan province (Chengdu, China: Dr. Hao, Southwest Minzu University).

[B24] Liu Y. H. Li B. B. Li K. R. He B. Zhang J. B. Pu X. F.. (2018). Molecular detection of *R. turanicus* and its eggs carrying *R. raoultii* in Southern Xinjiang. Scientia Agric. Sin. 51, 3020–3028. doi: 10.3864/j.issn.0578-1752.2018.15.017

[B25] Livengood J. Hutchinson M. L. Thirumalapura N. Tewari D. (2020). Detection of *Babesia*, *Borrelia*, *Anaplasma*, and *Rickettsia* spp. in Adult Black-Legged Ticks (*Ixodes scapularis*) from Pennsylvania, United States, with a Luminex Multiplex Bead Assay. Vector Borne Zoonotic Dis. 20, 406–411. doi: 10.1089/vbz.2019.2551 31976829

[B26] Mediannikov O. Matsumoto K. Samoylenko I. Drancourt M. Roux V. Rydkina E.. (2008). *Rickettsia raoultii* sp. nov., a spotted fever group *Rickettsia* associated with *Dermacentor* ticks in Europe and Russia. Int. J. Syst. Evol. Microbiol. 58, 1635–1639. doi: 10.1099/ijs.0.64952-0 18599708

[B27] Namgyal J. Couloigner I. Lysyk T. J. Dergousoff S. J. Cork S. C. (2020). Comparison of habitat suitability models for *Haemaphysalis longicornis* Neumann in North America to determine its potential geographic range. Int. J. Environ. Res. Public Health 17, 8285. doi: 10.3390/ijerph17218285 33182472 PMC7665130

[B28] Oteo J. A. Portillo A. Santibáñez S. Blanco J. R. Pérez-Martínez L. Ibarra V. (2006). Cluster of cases of human *Rickettsia felis* infection from southern europe (Spain) diagnosed by PCR. J. Clin. Microbiol. 44, 2669–2671. doi: 10.1128/JCM.00366-06 16825412 PMC1489505

[B29] Parola P. Paddock C. D. Socolovschi C. Labruna M. B. Mediannikov O. Kernif T.. (2013). Update on tick-borne rickettsioses around the world: a geographic approach. Clin. Microbiol. Rev. 26, 657–702. doi: 10.1128/CMR.00032-13 24092850 PMC3811236

[B30] Raoult D. Fournier P. E. Eremeeva M. Graves S. Kelly P. J. Oteo J. A.. (2005). Naming of *Rickettsiae* and rickettsial diseases. Ann. N Y Acad. Sci. 1063, 1–12. doi: 10.1196/annals.1355.002 16481485

[B31] Rar V. Livanova N. Tkachev S. Kaverina G. Tikunov A. Sabitova Y.. (2017). Detection and genetic characterization of a wide range of infectious agents in *Ixodes pavlovskyi* ticks in Western Siberia, Russia. Parasite Vector 10, 258. doi: 10.1186/s13071-017-2186-5 PMC5445278 28545549

[B32] Rydkina E. Roux V. Rudakov N. Gafarova M. Tarasevich I. Raoult D. (1999). New Rickettsiae in ticks collected in territories of the former Soviet Union. Emerg. Infect. Dis. 5, 811–814. doi: 10.3201/eid0506.990612 10603217 PMC2640811

[B33] Sayers E. W. Beck J. Bolton E. E. Bourexis D. Brister J. R. Canese K.. (2021). Database resources of the national center for biotechnology information. Nucleic Acids Res. 49, D10–D17. doi: 10.1093/nar/gkaa892 33095870 PMC7778943

[B34] Shpynov S. Fournier P. E. Rudakov N. Arsen’eva I. Granitov M. Tarasevich I.. (2009). Tick-borne rickettsiosis in the Altay region of Russia. Clin. Microbiol. Infect. 15, 313–314. doi: 10.1111/j.1469-0691.2008.02255.x 19438645

[B35] Spitalska E. Stefanidesova K. Kocianova E. Boldis V. (2012). *Rickettsia slovaca* and *Rickettsia raoultii* in *Dermacentor marginatus* and *Dermacentor reticulatus* ticks from Slovak Republic. Exp. Appl. Acarol 57, 189–197. doi: 10.1007/s10493-012-9539-8 22392435

[B36] Tang T. C. Liu C. C. Yuan D. B. Guo L. Hou W. Mo X.. (2019). Molecular detection of *Bartonella* infection in ixodid ticks collected from yaks in Shiqu County of Sichuan Province. Acta Agric. Zhejiangensis 31, 1066–1072. doi: 10.3969/j.issn.1004-1524.2019.07.05

[B37] Wang Y. Liu Z. Yang J. Chen Z. Liu J. Li Y.. (2012). *Rickettsia raoultii*-like bacteria in *Dermacentor* spp. ticks, Tibet, China. Emerg. Infect. Dis. 18, 1532–1534. doi: 10.3201/eid1809.120644 22931966 PMC3437703

[B38] Wei Q. Q. Guo L. P. Wang A. D. Mu L. M. Zhang K. Chen C. F.. (2015). The first detection of *Rickettsia aeschlimannii* and *Rickettsia massiliae* in *Rhipicephalus turanicus* ticks, in northwest China. Parasite Vector 8, 631. doi: 10.1186/s13071-015-1242-2 PMC4675064 26652857

[B39] Ybañez A. P. Matsumoto K. Kishimoto T. Inokuma H. (2012). Molecular analyses of a potentially novel *Anaplasma* species closely related to *Anaplasma phagocytophilum* detected in sika deer (*Cervus nippon yesoensis*) in Japan. Vet. Microbiol. 157, 232–236. doi: 10.1016/j.vetmic.2011.12.001 22204789

[B40] Yin X. Guo S. Ding C. Cao M. Kawabata H. Sato K.. (2018). Spotted fever group rickettsiae in inner Mongolia, China 2015-2016. Emerg. Infect. Dis. 24, 2105–2107. doi: 10.3201/eid2411.162094 30334715 PMC6200000

[B41] Zhang Y. Wen X. X. Xiao P. P. Fan X. L. Li M. Chahan B. Y. (2021). Molecular identification of *Theileria equi*, *Babesia caballi*, and *Rickettsia* in adult ticks from North of Xinjiang, China. Vet. Med. Sci. 7, 2219–2224. doi: 10.1002/vms3.v7.6 34448371 PMC8604137

[B42] Zhao L. Li J. Cui X. Jia N. Wei J. Xia L.. (2020). Distribution of *Haemaphysalis longicornis* and associated pathogens: analysis of pooled data from a China field survey and global published data. Lancet Planet Health 4, e320–e329. doi: 10.1016/S2542-5196(20)30145-5 32800150

[B43] Zheng W. Q. Xuan X. N. Fu R. L. Tao H. Y. Liu Y. Q. Liu X. Q.. (2018). Tick-Borne pathogens in *Ixodid* ticks from Poyang Lake Region, Southeastern China. Korean J. Parasitol. 56, 589–596. doi: 10.3347/kjp.2018.56.6.589 30630280 PMC6327193

[B44] Zhou S. Krzton A. Gao S. Guo C. Xiang Z. F. (2021). Effects of human activity on the habitat utilization of Himalayan marmot (*Marmota himalayana*) in Zoige wetland. Ecol. Evol. 11, 8957–8968. doi: 10.1002/ece3.v11.13 34257938 PMC8258216

