# Supplementary figures and images for: Pathogenetic identification in ticks and yaks from Zoige County, China
Source: Front Cell Infect Microbiol. 2024 Oct 21;14:1474519. doi: 10.3389/fcimb.2024.1474519 (PMC11532068; doi:10.3389/fcimb.2024.1474519)

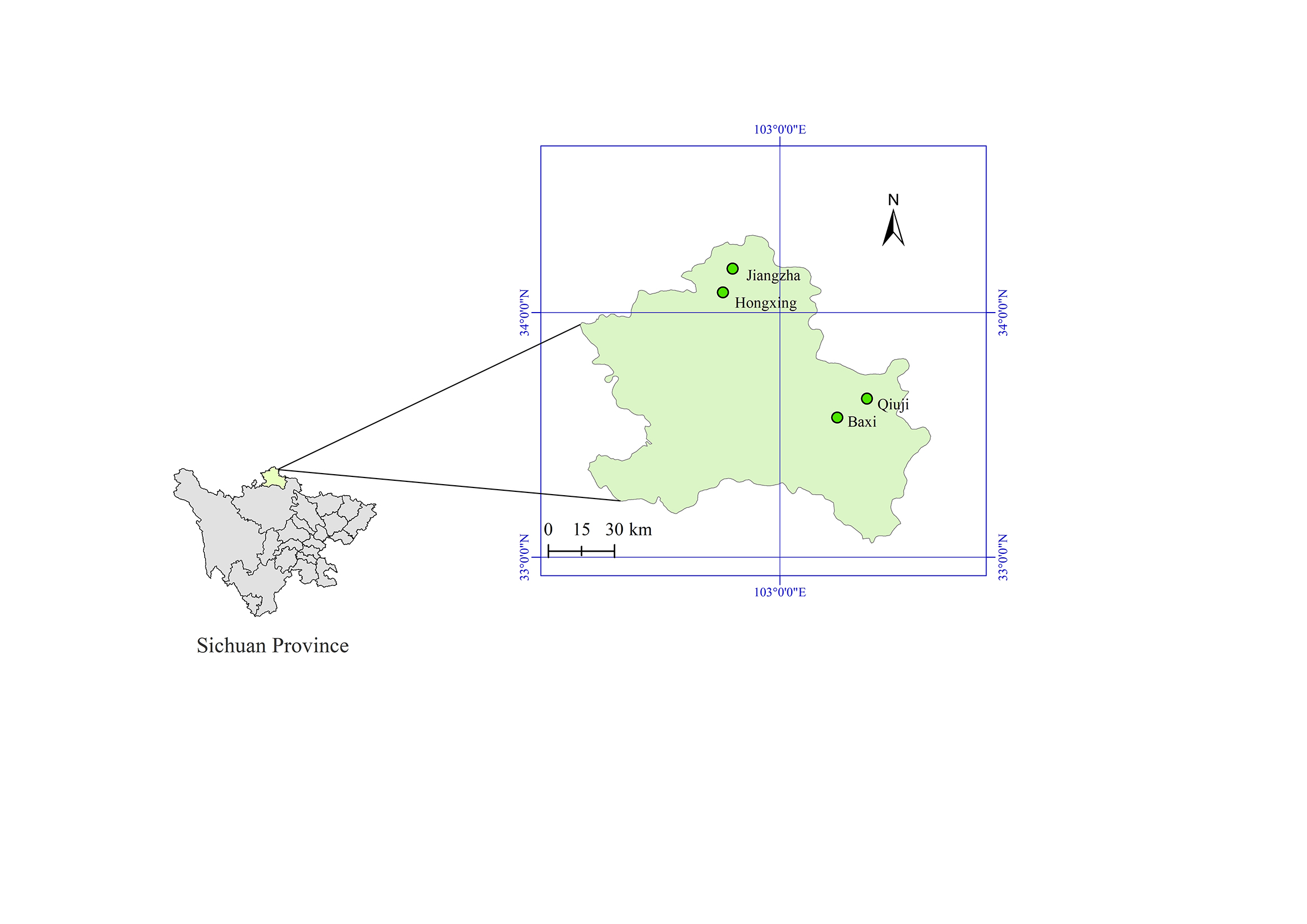

Supplement: Supplementary Figure 1 — Tick sampling information. [file Image1.jpeg]
